# Human Herpesviridae Methods of Natural Killer Cell Evasion

**DOI:** 10.1155/2012/359869

**Published:** 2012-07-08

**Authors:** Carl I. Odom, David C. Gaston, James M. Markert, Kevin A. Cassady

**Affiliations:** ^1^School of Medicine, University of Alabama at Birmingham, 1600 6th Avenue South, CHB 118C, Birmingham, AL 35233-1701, USA; ^2^Division of Neurosurgery, Department of Surgery, University of Alabama at Birmingham, 1530 3rd Avenue South, FOT 1060, Birmingham, AL 35294-3410, USA; ^3^Department of Cell, Developmental, and Integrative Biology, University of Alabama at Birmingham, 1530 3rd Avenue South, Birmingham, AL 35294-3410, USA; ^4^Division of Infectious Disease, Department of Pediatrics, University of Alabama at Birmingham, 600 7th Avenue South, CHB 118, Birmingham, AL 35233-1701, USA

## Abstract

Human herpesviruses cause diseases of considerable morbidity and mortality, ranging from encephalitis to hematologic malignancies. As evidence emerges about the role of innate immunity and natural killer (NK) cells in the control of herpesvirus infection, evidence of viral methods of innate immune evasion grows as well. These methods include interference with the ligands on infected cell surfaces that bind NK cell activating or inhibitory receptors. This paper summarizes the most extensively studied NK cell receptor/ligand pairs and then describes the methods of NK cell evasion used by all eight herpesviruses through these receptors and ligands. Although great strides have been made in elucidating their mechanisms, there is still a disparity between viruses in the amount of knowledge regarding innate immune evasion. Further research of herpesvirus innate immune evasion can provide insight for circumventing viral mechanisms in future therapies.

## 1. Introduction (Herpesviridae and Disease)

The human herpes family of viruses includes human cytomegalovirus (HCMV), Kaposi's sarcoma herpesvirus (KSHV), herpes simplex virus types 1 and 2 (HSV-1, 2), varicella zoster virus (VZV), Epstein-Barr virus (EBV), and human herpesvirus 6 and 7 (HHV6, 7). These viruses share similar characteristics: all contain linear double-stranded DNA, are enveloped, and undergo latent and lytic lifecycles. However, there are important differences between these viruses in terms of infection niche and immune evasion strategies for persistent infection.

Herpesviridae evasion of adaptive immune responses has been previously described [1–4]. This paper will focus on herpesvirus innate immune evasion, specifically viral evasion of the natural killer (NK) cells response. Reviews on broad interactions between viruses and NK cells can be found in references [5–8]. The role of NK cells in controlling herpes viral infections become apparent in consideration that multiple herpes infections have been documented in patients lacking NK cells [9] and evidence of NK activation during viral infection [10–13].

## 2. NK Cells and Activation

NK cells are important innate immune cells involved in the regulation of viral infection [14, 15]. They are a lymphocyte subset of the innate immune system that kills without prior exposure and sensitization to antigens via release of granzymes, perforin, TRAIL, and FAS ligand [16]. NK cells are regulated through surface receptor interactions with ligands expressed on stressed cells, such as virally infected or malignantly transformed cells. NK cells possess both activating and inhibitory cell surface receptors; it is the balance of ligand interactions with these receptors that determine NK cell activation. The structures, functions, and signaling mechanisms of these receptors and their ligands are comprehensively reviewed in references [16–21]. In addition to receptor-mediated regulation, cytokines induced during viral infection (IL-15, IL-12, IL-8, IFN-*α*, and IFN-*β*) can indirectly activate NK cells as well [6]. A summary of the receptors present on NK cells and associated ligands most relevant to immune evasion by human herpesviruses is provided below.

### 2.1. Activating Receptors and Ligands

#### 2.1.1. Natural Killer Group 2 Member D (NKG2D) Receptor

NKG2D is a receptor found prominently on NK cells that provides activation signals through the coreceptor DAP-10 upon ligand binding. The ligands that bind NKG2D include (1) the MHC-I-like molecules MHC-class-I-polypeptide-related sequence A (MICA) and B (MICB), (2) UL16 binding proteins (ULBP1–4 and 6), and (3) retinoic acid early transcript 1G (RAET1G). This interaction with multiple activating ligands is unique to NKG2D and does not occur with the other NK cell activating receptors [22, 23]. Investigators have proposed that this development of multiple activating ligands is a coevolutionary responses to viral or tumor pressure [22]. The structures of MICA and MICB are similar to MHC-I with alpha domains; however, they do not engage *β*2-microglobulin [24–26]. Surface expression of these ligands is normally absent or low on healthy cells and increases upon events of cellular stress such as viral infection, DNA damage, oxidative stress, and oncogenic stress [22, 27–30]. MICA is noted to have a large polymorphic distribution, with over 73 alleles identified [31]. A subset group of MICA alleles contains a frameshift mutation resulting in a premature stop codon and subsequent truncation of the cytoplasmic C-terminus. Interestingly, the MICA allele ∗008 encodes a truncated protein and is the most frequently distributed MICA allele in various populations across the world [32–39]. ULBP1–4 and RAET1 have alpha1 and alpha2 domains similar to MICA/B; however, unlike MICA/B, they do not contain alpha3 domains and their mRNA is expressed at low levels even in normal cells without corresponding surface expression [23, 40].

#### 2.1.2. Natural Cytotoxicity Receptors (NCRs)

The NCRs contain immunoglobulin (Ig)-like domains and include NKp30, NKp44, NKp46, and NKp80 [41, 42]. A role for NCRs has been implicated in the prognosis of leukemia [43, 44] and the recognition/killing of various solid tumors [45, 46]. Only NKp30 has a confirmed ligand, the tumor ligand B7-H6 [47, 48]. Additional ligands for the NCRs are unknown, although possible ligands have been identified and include nuclear factor BAT3 [49] and a number of viral hemagglutinin proteins and heparan sulfate structures [50, 51].

#### 2.1.3. DNAX Accessory Molecule-1 (DNAM-1)

DNAM-1 is a member of the Ig super family that recognizes CD112 (nectin-2) and the poliovirus receptor [17]. Similar to other activating receptors, there is expression of DNAM-1 ligands on various tumors resulting in DNAM-1-mediated killing alone or in concert with other receptors [52–56]. Aberrations in DNAM-1 expression or DNAM-1 expressing NK cells have also been linked to a variety of autoimmune diseases [57–59].

### 2.2. Inhibitory Receptors and Ligands

The primary inhibitory receptors include the killer Ig-like inhibitory receptors (KIRs) and CD94-NKG2A lectin-like inhibitory receptor. The KIRs and CD94-NKG2A bind to MHC-I molecules and diminish NK cell activation. There has been no evidence to date for their binding to MHC-II molecules. The receptor-ligand interactions for both KIRs and CD94-NKG2A are MHC-I isotype specific [60–62]. In accordance with the “Missing Self” hypothesis first proposed by Karre et al., the lack of MHC-I on target cells removes the inhibitory signals from NK cells, thus leading to unopposed activation [63, 64].

#### 2.2.1. Killer Ig-Like Inhibitory Receptors (KIRs)

The KIRs are members of the Ig superfamily that recognize MHC-I molecules of the HLA-C isotype on surrounding cells [17, 19]. The absence of HLA-C on tumor and virus infected cells can result in loss of NK cell inhibition [21, 65, 66].

#### 2.2.2. Leukocyte Ig-Like Receptor (LIR)-1

Like the KIRs, LIR-1 contains Ig domains and binds MHC-I, but with a lower affinity than other inhibitory receptors [17, 19]. LIR-1 expression is more variable on NK cells than other immune cells [17]. 

#### 2.2.3. CD94-NKG2A Lectin-Like Inhibitory Receptor

This receptor is a C-lectin-like heterodimer that recognizes MHC-I molecules of the HLA-E isotype. Ligation of HLA-E by CD94-NKG2A leads to inhibition of NK cells, yet HLA-E ligation can activate NK cells if CD94 is complexed to NKG2C or -E [17, 19]. Similar to KIRs, the CD94-NKG2A complex results in loss of NK cell inhibition in the absence of HLA-E. However, the uninhibited activity is not as strong as that mediated by KIRs [65].

## 3. Herpesviridae Methods of NK Cell Evasion

Human herpesviruses have evolved multiple mechanisms to dampen NK cell cytotoxicity, interacting with many of the factors influencing the balance of NK cell activation and inhibition. A summary of these mechanisms is provided in Table 1. A number of methods employed by human herpesviruses hinder the expression of NK cell ligands on infected cells. This method of immune evasion has been studied in different members of the herpesvirus family, defining marked similarities and stark differences between family members. Multiple mechanisms offset the indirect NK cell activation prompted by lack of MHC-I surface expression. As many human herpesviruses diminish MHC-I presentation of viral antigens to avoid detection by cytotoxic T lymphocytes, these mechanisms may offset the loss of NK cell inhibition from “Missing Self” [64, 67].

### 3.1. CMV

The HCMV product UL18 is an MHC-I homologue that binds the inhibitory NK cell receptor LIR, possibly as a means of increasing the inhibitory signal [80, 81]. However, inhibition via this ortholog is controversial [101–103]. CMV also encodes UL40, which stabilizes and promotes surface expression of the HLA-E isotype. This diminishes NK cell activation by increased ligation of the CD94-NKG2A receptor [82–84]. US11 targets HLA-A; US2 and US3 target HLA-A and HLA-G while sparing HLA-E; US6 targets HLA-A,G, and E for degradation to diminish cytotoxic T-cell detection [64, 67, 104].

In addition to inducing an inhibitory response, HCMV also suppresses activating ligands that bind NKG2D. HCMV UL16 binds MICB, ULBP1, and ULBP2 to sequester these proteins in the ER of infected cells but is unable to bind to RAET1G [23, 75–77, 105]. The crystal structure of UL16-MICB complex has been characterized in reference [106]. The HCMV protein UL142 blocks surface expression of some MICA alleles by interacting with the cytosolic carboxyl-terminal region of the transmembrane protein and retaining it in the golgi network, limiting surface expression of the activating ligand. HCMV UL142 cannot downmodulate truncated forms of MICA lacking the intracellular carboxy terminus. It is interesting that, of the >70 MICA allelic forms, the MICA∗008 truncated form is present in a majority of the population and may provide a selective advantage [68, 70]. There is also evidence that HCMV encodes the microRNA mIR-UL112 that decreases MICB production to escape NKG2D detection [78, 107].

In summary, HCMV has multiple means of NK cell ligand manipulation. UL18 is a mock MHC-I molecule that takes advantage of NK cell inhibitory receptors, while UL40 prolongs the inhibitory signals of actual host MHC-I molecules. UL16 retains NKG2D ligands (except MICA and ULBP3) to prevent activation, while UL142 downmodulates MICA in an allele-dependent manner. All of the methods of immune evasion used by CMV are more comprehensively reviewed in references [2, 108].

### 3.2. KSHV

KSHV encodes proteins that target MHC-I to prevent viral antigen presentation to T-lymphocytes as does HCMV; however, the molecular mechanisms differ. KSHV K3 and K5 are E3 ubiquitin ligases that transfer ubiquitin to the cytoplasmic tails of proteins [70, 85]. The K5 protein targets HLA-A and HLA-B for endocytosis from cell surfaces, while the K3 protein targets HLA-A, B, C, and E [85–87]. No interactions with HLA-G are known.

Like UL142 of CMV, the KSHV protein K5 also blocks surface expression of MICA and MICB but is unable to downregulate the MICA∗008 allele due to the absence of a cytoplasmic tail and lysine ubiquitin sites [71]. Ubiquitinated MICA proteins are endocytosed from the infected cell surface and retained in cytoplasmic vesicles without increased degradation [71]. K5-mediated downmodulation protects infected cells from NK cell cytotoxicity [71]. K5 also downmodulates the activating ligands B7-2, AICL, and ICAM-1 by a similar mechanism [71, 109]. In contrast to acute lytic infection, chronic infection with KSHV results in higher levels of MHC-I, MICA/B, PVR, and CD112 expression [110]. Akin to HCMV, KSHV encodes the microRNA miRK-12-7 inhibiting MICB expression [79]. Additionally, KSHV has been reported to infect NK cells, leading to downmodulation of the activating NCRs and NKG2D receptors on NK cell surfaces [110].

To summarize, KSHV is similar to CMV in that both viruses encode proteins downmodulating MHC-I and MICA/B. The mechanism by which KSHV proteins function diverges from HCMV proteins in that KSHV K3 and K5 ubiquinate and promote endocytosis of targeted proteins, whereas HCMV UL142 and UL16 prevent protein maturation and surface expression. Similar to HCMV UL142, KSHV K5 downmodulation of MICA is allele dependent. The mechanistic basis is the absence of ubiquitin sites in the truncated cytoplasmic tail. To date, there is no evidence of KSHV mechanisms affecting the expression of the ULBP or RAET ligands. KSHV specific immune evasion is reviewed in more detail in [70, 111].

### 3.3. HSV-1 and 2

The exact mechanisms by which HSV-1 modulates NK cell inhibitory and activating ligands are less studied than those for HCMV and KSHV. The HSV-1 and 2 US12 gene product (infected cell protein 47, ICP47) downmodulates MHC-I surface expression by suppressing MHC-I transport from the ER [64, 72]. ICP47 binds to the transporter associated with antigen presentation (TAP) and in doing so inhibits MHC-I antigen loading and expression of antigenic peptides generated by proteasomal degradation that then translocate from the cytosol to the ER lumen [88–90, 112]. Cells engineered to express ICP47 failed to express antigenic peptides [90]. Interestingly, HSV-1 induces expression of certain HLA-G isoforms while decreasing the surface expression of others [113].

The consequences of MHC-I downmodulation on NK cell recognition of HSV-1 and HSV-2 infected cells are controversial. Studies using antibody blocking of KIRs and MHC-I in conjunction with exogenous ICP47 expression suggest that the protective properties of MHC-I via KIR inhibitory signaling are rendered ineffective upon infection with HSV-1 [64]. However, some studies utilizing ICP47 deleted recombinant HSV-1 and anti-KIR antibodies suggest that NK cell inhibitory effects of MHC-I molecules are not significant enough alone to diminish cytotoxicity and that the viral product ICP47 is not necessary in inducing susceptibility to NK cell killing [73]. There are yet other findings that suggest a qualitative change in MHC-I molecules, such as the binding site shape presented to NK cells during HSV infection rather than the quantity of molecules presented, may contribute to NK recognition and killing [114].

There are few studies examining the influence of HSV-1 and HSV-2 infection upon the NK cell activating ligands. Experiments utilizing HSV-1 recombinants deleted of all IE genes except for ICP0 were able to induce NK cell induced lysis of human fibroblasts. Fibroblasts expressing ICP0 yielded similar results [73]. Infection with these recombinants also demonstrated an upregulation of unknown ligands binding to NCRs with cytotoxicity dependent upon their presence, yet this only occurred at low multiplicities of infection (MOIs). ICP0-independent mechanisms were reported at higher MOIs [73]. In contrast, studies of HSV effects on NKG2D ligands demonstrated decreased surface expression of MICA in HeLa and U373 cells infected with HSV-1 or HSV-2 with no difference in total protein levels [72]. Interestingly, this HSV-mediated downmodulation of MICA occurs with both the full-length and the truncated protein encoded by the MICA∗008 allele. This diverges significantly from the inability of HCMV and KSHV to down-modulate truncated MICA variants. Down-modulation of MICA was reported with ICP0 deleted recombinant HSV but not with PAA blocking of late gene expression, suggestive that this phenomenon is dependent on late-gene expression [72].

These studies together suggest HSV-1 and HSV-2 employ both early and late gene modulation of NK activating ligands, each with potentially different consequences for virus-infected cells. ICP0 might be sufficient to trigger NK cell cytotoxicity at low MOIs through upregulation of NCR ligands, which would be deleterious for virus survival. Yet at higher MOIs mechanisms other than ICP0 contribute to infected cell susceptibility. MICA downmodulation was shown to be independent of ICP0 expression and may be caused by late gene products. As posited by Schepis et al., HSV-1 may cause infected cells to be particularly susceptible to NK cell-mediated killing early in infection due to ICP0 upregulation of NCR ligands while attempting immunoevasion later in infection by NKG2D ligand and MHC-I downregulation [72]. Although HSV-1 and HSV-2 microRNAs have been documented, none have been found to interfere with NK cell pathways.

### 3.4. EBV

As with the previously described human herpesviruses, EBV has methods of interfering with MHC-I to prevent presentation of viral antigens to cytotoxic T cells. During active B-cell infection, EBV expresses a viral homolog of interleukin-10 (vIL-10) [91] and BNLF2a, a lytic-phase viral protein [92, 93]; both have been reported to downregulate the expression of TAP and in turn decrease surface MHC-I. BILF1 is a protein also expressed during lytic EBV infection that mediates both increased endocytosis/degradation and decreased exocytosis/presentation of MHC-I [94]. Downmodulation of all isotypes of MHC-I during EBV lytic infection, as well as subsequent decrease in inhibitory binding to KIRs and CD94-NKG2A, results in increased sensitivity to NK cell-mediated killing [115]. Instead of downregulation of activating ligands to offset this decrease in inhibition, the same studies found an increase in ULBP1 and CD112 expression that contributed to NK cell activation [115]. The only reported mechanism to possibly offset this indirect NK cell activation is a microRNA (miR-BART2-5p) inhibiting MICB expression [79]. EBV may possibly interfere with NKG2D activation through down-modulation of the NKG2D receptor itself via indoleamine-2, 3-dioxygenase metabolites, although the functional consequences have yet to be reported [116]. Similar to KSHV, EBV can infect NK cells, causing aberrantly high expression of the inhibitory CD94-NKG2A receptor but diminished expression of the KIRs [117].

### 3.5. HHV-6 and HHV-7

The involvement of NK cells in the control of HHV-6 and HHV-7 infection has been documented through studies of IL-2 and IL-15 enhancement of cytotoxicity [118–120]. However, the only documented HHV-7 protein involved in NK cell ligand modulation is U21, a transmembrane protein capable of downmodulating both MHC-I and MICA/B. U21 binds and redirects MHC-I trafficking to lysosomal compartments most similarly to HCMV MHC-I interfering proteins [96–98]. The effect on NK cell killing through this method has not been established. U21 also binds to ULBP1 for redirection to lysosomes and decreases surface MICA and MICB by an undefined mechanism that results in decreased NK cell cytotoxicity [74]. The A and B variants of HHV-6 express proteins analogous to U21 that also bind MHC-I for lysosome redirection [95], but the effect on NK cells or identification of mechanisms affecting activating ligands has not been established.

### 3.6. VZV

Although NK cells have long been implicated in the control of VZV infection [121–123], specific interactions with infected cells through NK cell receptors have not been extensively studied. VZV downregulates MHC-I on infected cell surfaces via the viral protein kinase ORF66, leading to retention of MHC-I molecules in the golgi [99, 100]. However, any functional consequences of ORF66 on NK cell recognition and killing have not been demonstrated. Likewise, no methods of VZV interference with NK cell activating ligands have been reported to date.

## 4. Conclusion

Human herpesviruses possess multiple mechanisms for evading both innate and adaptive immune responses. A summary of NK cell receptors, their ligands, and viral mechanisms interfering with each is provided in Table 1. A primary point of interest is the diversity of NK cell evasion mechanisms employed by human herpesviruses. Other than mechanisms shared by the highly similar HSV-1 and HSV-2, the human herpesviruses have evolved different mechanisms for subverting the immune response. It is also notable that these immunoevasion mechanisms do not group with herpesvirus subfamilies. For example, HCMV (a betaherpesvirus) and KSHV (a gammaherpesvirus) both encode microRNA's targeting MICB yet downmodulate MICA via different mechanisms. The NK cell evasion mechanisms are unique to each human herpesvirus likely reflecting selection pressures encountered in the various infection niches occupied by the viruses. The lack of well-defined mechanisms of NK cell immunoevasion by given herpesviruses (i.e., HSV and VZV) is puzzling. There is well-documented persistence of HSV in patients with NK cell defects and the importance of NK cell involvement in the control of disease. Continuing research will likely reveal as yet unknown mechanisms of immunoevasion by the alpha herpesviruses.

Human herpesviruses cause substantial morbidity and mortality. HSV-1 is regarded as one of the most common causes of viral encephalitis, an infection carrying significant risk of mortality [124, 125]. EBV, HCMV, and KSHV infections have the potential to not only cause severe manifestations during acute infection, but also the development of hematologic or solid malignancies [126, 127]. A variety of herpesviruses also cause cutaneous and ocular infections with potential for life-long morbidity [128–130]. Thorough knowledge of specific viral immune evasion mechanisms may provide avenues for developing more effective therapies against disease related to human herpesviruses. Understanding NK cell evasion may improve oncolytic herpesvirus therapies for cancer [131–133]. Insight into viral abilities to evade the immune system may also yield better markers for clinical prognosis and monitoring of active and latent infection [117, 134]. Continued research into these mechanisms of NK cell evasion will not only deepen basic understandings of human herpesviruses but may also serve to ultimately alleviate disease burden and guide strategies for clearance of persistent infection in immunocompromised patients.

## Figures and Tables

**Table 1:** Summary of known interactions between NK cell receptors, ligands, and herpesvirus immunoevasins.

Major receptors	Ligand	Virus	Immunoevasin	Mechanism	References
Activating					

	MICA	HCMV	UL142	Internal retention	[68, 69]
		KSHV	K5	Ubiquitination/sequestration	[70, 71]
		HSV	?	?	[72, 73]
		HHV-7	U21	?	[74]

		HCMV	UL16	Internal retention	[23, 75–77]
		HCMV	miR-UL112	Translational downregulation	[78]
NKG2D		KSHV	K5	Ubiquitination/sequestration	[70, 71]
	MICB	KSHV	miRK12-7	Translational downregulation	[79]
		HSV	?	?	[72, 73]
		EBV	miR-BART2-5p	Translational downregulation	[79]
		HHV-7	U21	?	[74]

		HCMV	UL16	Internal retention	[23, 75–77]
	ULBP1–4	HSV	?	?	[72]
		HHV-7	U21	Lysosomal degradation	[74]

NCRs	AICL	KSHV	K5		[71]

DNAM-1	PVR	?	?		
	CD112	?	?		

Inhibitory					

		HCMV	UL18	MHC-I homologue	[80, 81]
		HCMV	UL40	Signal prolongation	[82–84]
		HCMV	US2, US3, US6, US11	Retention/degradation	[64, 67]
		KSHV	K5, K3	Endocytosis	[85–87]
		HSV	ICP47	TAP interference	[88–90]
LIR-1, KIRs, CD94/NKG2A	MHC-I	EBV	vIL-10	IL-10 homolog	[91]
		EBV	BNLF2a	TAP interference	[92, 93]
		EBV	BILF1	Endocytosis/degradation	[94]
		HHV-6	U21 analogues	Lysosomal degradation	[95]
		HHV-7	U21	Lysosomal degradation	[96–98]
		VZV	ORF66	Internal retention	[99, 100]

## References

[1] Chan T, Barra NG, Lee AJ, Ashkar AA (2011). Innate and adaptive immunity against herpes simplex virus type 2 in the genital mucosa. *Journal of Reproductive Immunology*.

[2] Jackson SE, Mason GM, Wills MR (2011). Human cytomegalovirus immunity and immune evasion. *Virus Research*.

[3] Coscoy L (2007). Immune evasion by Kaposi’s sarcoma-associated herpesvirus. *Nature Reviews Immunology*.

[4] Wiertz EJ, Devlin R, Collins HL, Ressing ME (2007). Herpesvirus interference with major histocompatibility complex class II-restricted T-cell activation. *Journal of Virology*.

[5] Jonjić S, Babić M, Polić B, Krmpotić A (2008). Immune evasion of natural killer cells by viruses. *Current Opinion in Immunology*.

[6] Lanier LL (2008). Evolutionary struggles between NK cells and viruses. *Nature Reviews Immunology*.

[7] Groth A, Klöss S, Pogge Von Strandmann E, Koehl U, Koch J (2011). Mechanisms of tumor and viral immune escape from natural killer cell-mediated surveillance. *Journal of Innate Immunity*.

[8] Lisnić VJ, Krmpotić A, Jonjić S (2010). Modulation of natural killer cell activity by viruses. *Current Opinion in Microbiology*.

[9] Biron CA, Byron KS, Sullivan JL (1989). Severe herpesvirus infections in an adolescent without natural killer cells. *The New England Journal of Medicine*.

[10] Fitzgerald PA, Mendelsohn M, Lopez C (1985). Human natural killer cells limit replication of herpes simplex virus type 1 in vitro. *Journal of Immunology*.

[11] Paya CV, Schoon RA, Leibson PJ (1990). Alternative mechanisms of natural killer cell activation during herpes simplex virus infection. *Journal of Immunology*.

[12] Fitzgerald-Bocarsly P, Howell DM, Pettera L, Tehrani S, Lopez C (1991). Immediate-early gene expression is sufficient for induction of natural killer cell-mediated lysis of herpes simplex virus type 1-infected fibroblasts. *Journal of Virology*.

[13] Lopez C, Kirkpatrick D, Fitzgerald PA (1982). Studies of the cell lineage of the effector cells that spontaneously lyse HSV-1 infected fibroblasts (NK(HSV-1)). *Journal of Immunology*.

[14] Marras F, Bozzano F, De Maria A (2011). Involvement of activating NK cell receptors and their modulation in pathogen immunity. *Journal of Biomedicine and Biotechnology*.

[15] Lee SH, Biron CA (2010). Here today—not gone tomorrow: roles for activating receptors in sustaining NK cells during viral infections. *European Journal of Immunology*.

[16] Farag SS, Caligiuri MA (2006). Human natural killer cell development and biology. *Blood Reviews*.

[17] Lanier LL (2005). NK cell recognition. *Annual Review of Immunology*.

[18] Bryceson YT, Chiang SCC, Darmanin S (2011). Molecular mechanisms of natural killer cell activation. *Journal of Innate Immunity*.

[19] Joyce MG, Sun PD (2011). The structural basis of ligand recognition by natural killer cell receptors. *Journal of Biomedicine and Biotechnology*.

[20] Zafirova B, Wensveen FM, Gulin M, Polić B (2011). Regulation of immune cell function and differentiation by the NKG2D receptor. *Cellular and Molecular Life Sciences*.

[21] Purdy AK, Campbell KS (2009). Natural killer cells and cancer: regulation by the killer cell ig-like receptors (KIR). *Cancer Biology and Therapy*.

[22] Eagle RA, Trowsdale J (2007). Promiscuity and the single receptor: NKG2D. *Nature Reviews Immunology*.

[23] Raulet DH (2003). Roles of the NKG2D immunoreceptor and its ligands. *Nature Reviews Immunology*.

[24] Li P, Willie ST, Bauer S, Morris DL, Spies T, Strong RK (1999). Crystal structure of the MHC class I homolog MIC-A, a *γδ* T cell ligand. *Immunity*.

[25] Li P, Morris DL, Willcox BE, Steinle A, Spies T, Strong RK (2001). Complex structure of the activating immunoreceptor NKG2D and its MHC class I-like ligand MICA. *Nature Immunology*.

[26] Holmes MA, Li P, Petersdorf EW, Strong RK (2002). Structural studies of allelic diversity of the MHC class I homolog MIC-B, a stress-inducible ligand for the activating immunoreceptor NKG2D. *Journal of Immunology*.

[27] Moretta L, Bottino C, Pende D, Castriconi R, Mingari MC, Moretta A (2006). Surface NK receptors and their ligands on tumor cells. *Seminars in Immunology*.

[28] Stern-Ginossar N, Mandelboim O (2009). An integrated view of the regulation of NKG2D ligands. *Immunology*.

[29] Gasser S, Orsulic S, Brown EJ, Raulet DH (2005). The DNA damage pathway regulates innate immune system ligands of the NKG2D receptor. *Nature*.

[30] Yamamoto K, Fujiyama Y, Andoh A, Bamba T, Okabe H (2001). Oxidative stress increases MICA and MICB gene expression in the human colon carcinoma cell line (CaCo-2). *Biochimica et Biophysica Acta*.

[31] Frigoul A, Lefranc M-P, Pandalai SG (2005). MICA: standardized IMGT allele nomenclature, polymorphisms and diseases. *Recent Research Developments in Human Genetics*.

[32] Petersdorf EW, Shuler KB, Longton GM, Spies T, Hansen JA (1999). Population study of allelic diversity in the human MHC class I-related MIC-A gene. *Immunogenetics*.

[33] Zhu F, Zhao H, He Y (2009). Distribution of MICA diversity in the Chinese Han population by polymerase chain reaction sequence-based typing for exons 2-6. *Tissue Antigens*.

[34] Marin MLC, Savioli CR, Yamamoto JH, Kalil J, Goldberg AC (2004). MICA polymorphism in a sample of the São Paulo population, Brazil. *European Journal of Immunogenetics*.

[35] Gao X, Single RM, Karacki P, Marti D, O’Brien SJ, Carrington M (2006). Diversity of MICA and linkage disequilibrium with HLA-B in two North American populations. *Human Immunology*.

[36] Lucas D, Campillo JA, López-Hernández R (2008). Allelic diversity of MICA gene and MICA/HLA-B haplotypic variation in a population of the Murcia region in southeastern Spain. *Human Immunology*.

[37] Tian W, Boggs DA, Uko G (2003). MICA, HLA-B haplotypic variation in five population groups of sub-Saharan African ancestry. *Genes and Immunity*.

[38] Cambra A, Muñoz-Saá I, Crespí C (2009). MICA-HLA-B haplotype diversity and linkage disequilibrium in a population of Jewish descent from Majorca (the Balearic Islands). *Human Immunology*.

[39] Ďurmanová V, Tirpakova J, Stuchlikova M (2011). Characterization of MICA gene polymorphism of HLA complex in the Slovak population. *Annals of Human Biology*.

[40] Cosman D, Müllberg J, Sutherland CL (2001). ULBPs, novel MHC class I-related molecules, bind to CMV glycoprotein UL16 and stimulate NK cytotoxicity through the NKG2D receptor. *Immunity*.

[41] Bottino C, Biassoni R, Millo R, Moretta L, Moretta A (2000). The human natural cytotoxicity receptors (NCR) that induce HLA class I-independent NK cell triggering. *Human Immunology*.

[42] Vitale M, Falco M, Castriconi R (2001). Identification of NKp80, a novel triggering molecule expressed by human NK cells. *European Journal of Immunology*.

[43] Fauriat C, Just-Landi S, Mallet F (2007). Deficient expression of NCR in NK cells from acute myeloid leukemia: Evolution during leukemia treatment and impact of leukemia cells in NCR dull phenotype induction. *Blood*.

[44] Costello RT, Knoblauch B, Sanchez C, Mercier D, Le Treut T, Sébahoun G (2012). Expression of natural killer cell activating receptors in patients with chronic lymphocytic leukaemia. *Immunology*.

[45] Glasner A, Ghadially H, Gur C (2012). Recognition and prevention of tumor metastasis by the NK receptor NKp46/NCR1. *Journal of Immunology*.

[46] Morgado S, Sanchez-Correa B, Casado JG (2011). NK cell recognition and killing of melanoma cells is controlled by multiple activating receptor-ligand interactions. *Journal of Innate Immunity*.

[47] Li Y, Wang Q, Mariuzza RA (2011). Structure of the human activating natural cytotoxicity receptor NKp30 bound to its tumor cell ligand B7-H6. *Journal of Experimental Medicine*.

[48] Kaifu T, Escalière B, Gastinel LN, Vivier E, Baratin M (2011). B7-H6/NKp30 interaction: a mechanism of alerting NK cells against tumors. *Cellular and Molecular Life Sciences*.

[49] Pogge von Strandmann E, Simhadri VR, von Tresckow B (2007). Human leukocyte antigen-B-associated transcript 3 is released from tumor vells and engages the NKp30 receptor on natural killer cells. *Immunity*.

[50] Hecht ML, Rosental B, Horlacher T (2009). Natural cytotoxicity receptors NKp30, NKp44 and NKp46 bind to different heparan sulfate/heparin sequences. *Journal of Proteome Research*.

[51] Jarahian M, Watzl C, Fournier P (2009). Activation of natural killer cells by Newcastle disease virus hemagglutinin-neuraminidase. *Journal of Virology*.

[52] Carlsten M, Björkström NK, Norell H (2007). DNAX accessory molecule-1 mediated recognition of freshly isolated ovarian carcinoma by resting natural killer cells. *Cancer Research*.

[53] Castriconi R, Dondero A, Corrias MV (2004). Natural killer cell-mediated killing of freshly isolated neuroblastoma cells: Critical role of DNAX accessory molecule-1-poliovirus receptor interaction. *Cancer Research*.

[54] Chan CJ, Andrews DM, McLaughlin NM (2010). DNAM-1/CD155 interactions promote cytokine and NK cell-mediated suppression of poorly immunogenic melanoma metastases. *Journal of Immunology*.

[55] Lakshmikanth T, Burke S, Ali TH (2009). NCRs and DNAM-1 mediate NK cell recognition and lysis of human and mouse melanoma cell lines in vitro and in vivo. *Journal of Clinical Investigation*.

[56] Zhang Z, Su T, He L (2012). Identification and functional analysis of ligands for natural killer cell activating receptors in colon carcinoma. *The Tohoku journal of experimental medicine*.

[57] Huang Z, Fu B, Zheng SG (2011). Involvement of CD226^+^ NK cells in immunopathogenesis of systemic lupus erythematosus. *Journal of Immunology*.

[58] Maiti AK, Kim-Howard X, Viswanathan P (2010). Non-synonymous variant (Gly307Ser) in CD226 is associated with susceptibility to multiple autoimmune diseases. *Rheumatology*.

[59] Tjon JML, Kooy-Winkelaar YMC, Tack GJ (2011). DNAM-1 mediates epithelial cell-specific cytotoxicity of aberrant intraepithelial lymphocyte lines from refractory celiac disease type II patients. *Journal of Immunology*.

[60] Wagtmann N, Rajagopalan S, Winter CC, Peruzzi M, Long EO (1995). Killer cell inhibitory receptors specific for HLA-C and HLA-B identified by direct binding and by functional transfer. *Immunity*.

[61] Braud VM, Allan DSJ, O’Callaghan CA (1998). HLA-E binds to natural killer cell receptors CD94/NKG2A, B and C. *Nature*.

[62] Borrego F, Ulbrecht M, Weiss EH, Coligan JE, Brooks AG (1998). Recognition of human histocompatibility leukocyte antigen (HLA)-E complexed with HLA class I signal sequence-derived peptides by CD94/NKG2 confers protection from natural killer cell-mediated lysis. *Journal of Experimental Medicine*.

[63] Karre K, Ljunggren HG, Piontek G, Kiessling R (1986). Selective rejection of H-2-deficient lymphoma variants suggests alternative immune defence strategy. *Nature*.

[64] Huard B, Früh K (2000). A role for MHC class I down-regulation in NK cell lysis of herpes virus-infected cells. *European Journal of Immunology*.

[65] Yu J, Heller G, Chewning J, Kim S, Yokoyama WM, Hsu KC (2007). Hierarchy of the human natural killer cell response is determined by class and quantity of inhibitory receptors for self-HLA-B and HLA-C ligands. *Journal of Immunology*.

[66] Van Bergen J, Thompson A, Retière C, Trowsdale J, Koning F (2009). Cutting edge: killer Ig-like receptors mediate “missing self” recognition in vivo. *Journal of Immunology*.

[67] Lin A, Xu H, Yan W (2007). Modulation of HLA expression in human cytomegalovirus immune evasion. *Cellular & Molecular Immunology*.

[68] Chalupny NJ, Rein-Weston A, Dosch S, Cosman D (2006). Down-regulation of the NKG2D ligand MICA by the human cytomegalovirus glycoprotein UL142. *Biochemical and Biophysical Research Communications*.

[69] Ashiru O, Bennett NJ, Boyle LH, Thomas M, Trowsdale J, Wills MR (2009). NKG2D ligand MICA is retained in the cis-Golgi apparatus by human cytomegalovirus protein UL142. *Journal of Virology*.

[70] Thomas M, Wills M, Lehner PJ (2008). Natural killer cell evasion by an E3 ubiquitin ligase from Kaposi’s sarcoma-associated herpesvirus. *Biochemical Society Transactions*.

[71] Thomas M, Boname JM, Field S (2008). Down-regulation of NKG2D and NKp80 ligands by Kaposi’s sarcoma-associated herpesvirus K5 protects against NK cell cytotoxicity. *Proceedings of the National Academy of Sciences of the United States of America*.

[72] Schepis D, D’Amato M, Studahl M, Bergström T, Kärre K, Berg L (2009). Herpes simplex virus infection downmodulates NKG2D ligand expression. *Scandinavian Journal of Immunology*.

[73] Chisholm SE, Howard K, Gómez MV, Reyburn HT (2007). Expression of ICP0 is sufficient to trigger natural killer cell recognition of herpes simplex virus-infected cells by natural cytotoxicity receptors. *Journal of Infectious Diseases*.

[74] Schneider CL, Hudson AW (2011). The human herpesvirus-7 (HHV-7) U21 immunoevasin subverts NK-mediated cytoxicity through modulation of MICA and MICB. *PLoS Pathogens*.

[75] Welte SA, Sinzger C, Lutz SZ (2003). Selective intracellular retention of virally induced NKG2D ligands by the human cytomegalovirus UL16 glycoprotein. *European Journal of Immunology*.

[76] Wu J, Chalupny NJ, Manley TJ, Riddell SR, Cosman D, Spies T (2003). Intracellular retention of the MHC class I-related chain B ligand of NKG2D by the human cytomegalovirus UL16 glycoprotein. *Journal of Immunology*.

[77] Dunn C, Chalupny NJ, Sutherland CL (2003). Human cytomegalovirus glycoprotein UL16 causes intracellular sequestration of NKG2D ligands, protecting against natural killer cell cytotoxicity. *Journal of Experimental Medicine*.

[78] Stern-Ginossar N, Elefant N, Zimmermann A (2007). Host immune system gene targeting by a viral miRNA. *Science*.

[79] Nachmani D, Stern-Ginossar N, Sarid R, Mandelboim O (2009). Diverse herpesvirus microRNAs target the stress-induced immune ligand MICB to escape recognition by natural killer cells. *Cell Host and Microbe*.

[80] Reyburn HT, Mandelboim O, Valés-Gómez M, Davis DM, Pazmany L, Strominger JL (1997). The class I MHC homologue of human cytomegalovirus inhibits attack by natural killer cells. *Nature*.

[81] Yang Z, Bjorkman PJ (2008). Structure of UL18, a peptide-binding viral MHC mimic, bound to a host inhibitory receptor. *Proceedings of the National Academy of Sciences of the United States of America*.

[82] Tomasec P (2000). Surface expression of HLA-E, an inhibitor of natural killer cells, enhanced by human cytomegalovirus gpUL40. *Science*.

[83] Ulbrecht M, Martinozzi S, Grzeschik M (2000). Cutting edge: the human cytomegalovirus UL40 gene product contains a ligand for HLA-E and prevents NK cell-mediated lysis. *Journal of Immunology*.

[84] Wang ECY, McSharry B, Retiere C (2002). UL40-mediated NK evasion during productive infection with human cytomegalovirus. *Proceedings of the National Academy of Sciences of the United States of America*.

[85] Ishido S, Wang C, Lee BS, Cohen GB, Jung JU (2000). Downregulation of major histocompatibility complex class I molecules by Kaposi’s sarcoma-associated herpesvirus K3 and K5 proteins. *Journal of Virology*.

[86] Haque M, Ueda K, Nakano K (2001). Major histocompatibility complex class I molecules are down-regulated at the cell surface by the K5 protein encoded by Kaposi’s sarcoma-associated herpesvirus/human herpesvirus-8. *Journal of General Virology*.

[87] Bartee E, Mansouri M, Nerenberg BTH, Gouveia K, Früh K (2004). Downregulation of major histocompatibility complex class I by human ubiquitin ligases related to viral Immune evasion proteins. *Journal of Virology*.

[88] Hill AB, Barnett BC, McMichael AJ, McGeoch DJ (1994). HLA class I molecules are not transported to the cell surface in cells infected with herpes simplex virus types 1 and 2. *Journal of Immunology*.

[89] Hill A, Juovic P, York I (1995). Herpes simplex virus turns off the TAP to evade host immunity. *Nature*.

[90] York IA, Roop C, Andrews DW, Riddell SR, Graham FL, Johnson DC (1994). A cytosolic herpes simplex virus protein inhibits antigen presentation to CD8^+^ T lymphocytes. *Cell*.

[91] Sin S-H, Dittmer DP (2012). Cytokine homologs of human gammaherpesviruses. *Journal of Interferon and Cytokine Research*.

[92] Croft NP, Shannon-Lowe C, Bell AI (2009). Stage-specific inhibition of MHC class I presentation by the epstein-barr virus BNLF2a protein during virus lytic cycle. *PLoS Pathogens*.

[93] Horst D, Favaloro V, Vilardi F (2011). EBV protein BNLF2a exploits host tail-anchored protein integration machinery to inhibit TAP. *Journal of Immunology*.

[94] Zuo J, Quinn LL, Tamblyn J (2011). The Epstein-Barr virus-encoded BILF1 protein modulates immune recognition of endogenously processed antigen by targeting major histocompatibility complex class I molecules trafficking on both the exocytic and endocytic pathways. *Journal of Virology*.

[95] Glosson NL, Hudson AW (2007). Human herpesvirus-6A and -6B encode viral immunoevasins that downregulate class I MHC molecules. *Virology*.

[96] Glosson NL, Gonyo P, May NA (2010). Insight into the mechanism of human herpesvirus 7 U21-mediated diversion of class I MHC molecules to lysosomes. *Journal of Biological Chemistry*.

[97] May NA, Glosson NL, Hudson AW (2010). Human herpesvirus 7 U21 downregulates classical and nonclassical class I major histocompatibility complex molecules from the cell surface. *Journal of Virology*.

[98] Hudson AW, Blom D, Howley PM, Ploegh HL (2003). The ER-lumenal domain of the HHV-7 immunoevasin U21 directs class I MHC molecules to lysosomes. *Traffic*.

[99] Abendroth A, Lin I, Slobedman B, Ploegh H, Arvin AM (2001). Varicella-zoster virus retains major histocompatibility complex class I proteins in the golgi compartment of infected cells. *Journal of Virology*.

[100] Eisfeld AJ, Yee MB, Erazo A, Abendroth A, Kinchington PR (2007). Downregulation of class I major histocompatibility complex surface expression by varicella-zoster virus involves open reading frame 66 protein kinase-dependent and -independent mechanisms. *Journal of Virology*.

[101] Leong CC, Chapman TL, Bjorkman PJ (1998). Modulation of natural killer cell cytotoxicity in human cytomegalovirus infection: The role of endogenous class I major histocompatibility complex and a viral class I homolog. *Journal of Experimental Medicine*.

[102] Cosman D, Fanger N, Borges L (1997). A novel immunoglobulin superfamily receptor for cellular and viral MHC class I molecules. *Immunity*.

[103] Wills MR, Ashiru O, Reeves MB (2005). Human cytomegalovirus encodes an MHC Class I-like molecule (UL142) that functions to inhibit NK cell lysis. *Journal of Immunology*.

[104] Llano M, Gumá M, Ortega M, Angulo A, López-Botet M (2003). Differential effects of US2, US6, and US11 human cytomegalovirus proteins of HLA class Ia and HLA-E expression: Impact on target susceptibility to NK cell subsets. *European Journal of Immunology*.

[105] Wittenbrink M, Spreu J, Steinle A (2009). Differential NKG2D binding to highly related human NKG2D ligands ULBP2 and RAET1G is determined by a single amino acid in the *α*2 domain. *European Journal of Immunology*.

[106] Müller S, Zocher G, Steinle A, Stehle T (2010). Structure of the HCMV UL16-MICB complex elucidates select binding of a viral immunoevasin to diverse NKG2D ligands. *PLoS Pathogens*.

[107] Colonna M, Jonjic S, Watzl C (2011). Natural killer cells: fighting viruses andmuch more. *Nature Immunology*.

[108] Mocarski ES (2004). Immune escape and exploitation strategies of cytomegaloviruses: impact on and imitation of the major histocompatibility system. *Cellular Microbiology*.

[109] Ishido S, Choi JK, Lee BS (2000). Inhibition of natural killer cell-mediated cytotoxicity by Kaposi’s sarcoma-associated herpesvirus K5 protein. *Immunity*.

[110] Dupuy S, Lambert M, Zucman D (2012). Human herpesvirus 8 (HHV8) sequentially shapes the NK cell repertoire during the course of asymptomatic infection and Kaposi sarcoma. *PLoS Pathogens*.

[111] Means RE, Choi JK, Nakamura H, Chung YH, Ishido S, Jung JU (2002). Immune evasion strategies of Kaposi’s sarcoma-associated herpesvirus. *Current Topics in Microbiology and Immunology*.

[112] Tomazin R, Van Schoot NEG, Goldsmith K (1998). Herpes simplex virus type 2 ICP47 inhibits human TAP but not mouse tap. *Journal of Virology*.

[113] Lafon M, Prehaud C, Megret F (2005). Modulation of HLA-G expression in human neural cells after neurotropic viral infections. *Journal of Virology*.

[114] Kaufman DS, Schoon RA, Leibson PJ (1992). Role for major histocompatibility complex class I in regulating natural killer cell-mediated killing of virus-infected cells. *Proceedings of the National Academy of Sciences of the United States of America*.

[115] Pappworth IY, Wang EC, Rowe M (2007). The switch from latent to productive infection in Epstein-Barr virus-infected B cells is associated with sensitization to NK cell killing. *Journal of Virology*.

[116] Song H, Park H, Kim J (2011). IDO metabolite produced by EBV-transformed B cells inhibits surface expression of NKG2D in NK cells via the c-Jun N-terminal kinase (JNK) pathway. *Immunology Letters*.

[117] Sawada A, Sato E, Koyama M (2006). NK-cell repertoire is feasible for diagnosing Epstein-Barr virus-infected NK-cell lymphoproliferative disease and evaluating the treatment effect. *American Journal of Hematology*.

[118] Kida K, Isozumi R, Ito M (2000). Killing of human herpes virus 6-infected cells by lymphocytes cultured with interleukin-2 or -12. *Pediatrics International*.

[119] Flamand L, Stefanescu I, Menezes J (1996). Human herpesvirus-6 enhances natural killer cell cytotoxicity via IL-15. *Journal of Clinical Investigation*.

[120] Atedzoe BN, Ahmad A, Menezes J (1997). Enhancement of natural killer cell cytotoxicity by the human herpesvirus-7 via IL-15 induction. *Journal of Immunology*.

[121] Tilden AB, Cauda R, Grossi CE (1986). Demonstration of NK cell-mediated lysis of varicella-zoster virus (VZV)-infected cells: characterization of the effector cells. *Journal of Immunology*.

[122] Ito M, Bandyopadhyay S, Matsumoto-Kobayashi M (1986). Interleukin 2 enhances natural killing of varicella-zoster virus-infected targets. *Clinical and Experimental Immunology*.

[123] Hayward AR, Herberger M, Lazslo M (1986). Cellular interactions in the lysis of varicella-zoster virus infected human fibroblasts. *Clinical and Experimental Immunology*.

[124] Rozenberg F, Deback C, Agut H (2011). Herpes simplex encephalitis: from virus to therapy. *Infectious Disorders Drug Targets*.

[125] Stahl J-P, Mailles A, Dacheux L, Morand P (2011). Epidemiology of viral encephalitis in 2011. *Medecine et Maladies Infectieuses*.

[126] Gantt S, Casper C (2011). Human herpesvirus 8-associated neoplasms: the roles of viral replication and antiviral treatment. *Current Opinion in Infectious Diseases*.

[127] Taylor GS, Blackbourn DJ (2011). Infectious agents in human cancers: lessons in immunity and immunomodulation from gammaherpesviruses EBV and KSHV. *Cancer Letters*.

[128] Chisholm C, Lopez L (2011). Cutaneous infections caused by herpesviridae: a review. *Archives of Pathology and Laboratory Medicine*.

[129] Al-Dujaili LJ, Clerkin PP, Clement C (2011). Ocular herpes simplex virus: how are latency, reactivation, recurrent disease and therapy interrelated?. *Future Microbiology*.

[130] Berardi A, Lugli L, Rossi C (2011). Neonatal herpes simplex virus. *Journal of Maternal-Fetal and Neonatal Medicine*.

[131] Fulci G, Breymann L, Gianni D (2006). Cyclophosphamide enhances glioma virotherapy by inhibiting innate immune responses. *Proceedings of the National Academy of Sciences of the United States of America*.

[132] Fulci G, Dmitrieva N, Gianni D (2007). Depletion of peripheral macrophages and brain microglia increases brain tumor titers of oncolytic viruses. *Cancer Research*.

[133] Wakimoto H, Fulci G, Tyminski E, Chiocca EA (2004). Altered expression of antiviral cytokine mRNAs associated with cyclophosphamide’s enhancement of viral oncolysis. *Gene Therapy*.

[134] Haedicke W, Ho FCS, Chott A (2000). Expression of CD94/NKG2A and killer immunoglobulin-like receptors in NK cells and a subset of extranodal cytotoxic T-cell lymphomas. *Blood*.

